# Can a reresection be avoided after initial *en bloc* resection for high-risk nonmuscle invasive bladder cancer? A systematic review and meta-analysis

**DOI:** 10.3389/fsurg.2022.849929

**Published:** 2022-09-14

**Authors:** Jiangnan Xu, Zhenyu Xu, HuMin Yin, Jin Zang

**Affiliations:** ^1^ Department of Urology, Yancheng First Hospital, Affiliated Hospital of Nanjing University Medical School, Yancheng, China; ^2^ Department of Urology, The First People’s Hospital of Yancheng, Yancheng, China; ^3^ Department of Urology, Kunshan Chinese Medicine Hospital Affiliated to Nanjing University of Chinese Medicine, Suzhou, China; ^4^ Department of Urology, The First Affiliated Hospital of Soochow University, Suzhou, China

**Keywords:** high-risk, nonmuscle-invasive bladder cancer, *en bloc* resection, reresection, systematic review and meta-analysis

## Abstract

**Background:**

This study aims to evaluate the effectiveness of *en bloc* resection for patients with nonmuscle invasive bladder cancer (NMIBC) and explore whether a reresection can be avoided after initial *en bloc* resection.

**Material and methods:**

We conducted research in PubMed, EMBASE, Cochrane Library, and Web of Science up to October 12, 2021, to identify studies on the second resection after initial *en bloc* resection of bladder tumor (ERBT). R software and the double arcsine method were used for data conversion and combined calculation of the incidence rate.

**Results:**

A total of 8 studies involving 414 participants were included. The rate of detrusor muscle in the ERBT specimens was 100% (95%CI: 100%–100%), the rate of tumor residual in reresection specimens was 3.2% (95%CI: 1.4%–5.5%), and the rate of tumor upstaging was 0.3% (95%CI: 0%–1.5%). Two articles compared the prognostic data of the reresection and non-reresection groups after the initial ERBT. We found no significant difference in the 1-year recurrence-free survival (RFS) rate (OR = 1.44, 95%CI: 0.67–3.09, *P* = 0.35) between the two groups nor in the rate of tumor recurrence (OR = 0.72, 95%CI: 0.44–1.18, *P* = 0.2) or progression (OR = 0.98, 95%CI: 0.33–2.89, *P* = 0.97) at the final follow-up.

**Conclusions:**

ERBT can almost completely remove the detrusor muscle of the tumor bed with a very low postoperative tumor residue and upstaging rate. For high-risk NMIBC patients, an attempt to appropriately reduce the use of reresection after ERBT seems to be possible.

## Introduction

At present, transurethral resection of the bladder tumor (TURBT) combined with postoperative intravesical instillation is the gold standard for the treatment of nonmuscle invasive bladder cancer (NMIBC) (1). However, due to piecemeal resection, traditional TURBT has a high tumor residual rate, making it difficult to provide accurate pathological staging (2, 3). For accurate staging and detection of tumor residue, reresection is recommended for patients with high-risk NMIBC, although it significantly increases the complication risk and financial stress (1, 4).

Different from traditional TURBT, as a new strategy, transurethral *en bloc* resection of bladder tumor (ERBT) can theoretically wholly remove the bladder tumor and even achieve a 100% detrusor muscle (DM) presence rate. Several recent studies also confirmed that detrusor muscle was present in above 95% of ERBT specimens (5–9). Some previous studies showed that the presence rate of DM was closely related to recurrence and could be a surrogate marker of resection quality (10–12). Although the latest study by Mastroianni et al. showed that the absence of DM has no impact on tumor recurrence, the high DM presence rate and tumor tissue integrity could provide a significant advantage in tumor staging (13, 14). Xu et al. performed reresection on high-risk NMIBC patients who underwent initial ERBT. The results showed that the residual tumor rate and tumor progression rate were only 5.9% and 3.9%, respectively. Moreover, they found that reresection did not seem to improve the prognosis of these patients (5). Given the advantages of ERBT, is it possible to reduce the need for a reresection in high-risk NMIBC patients after initial ERBT?

To answer this question, we conducted a meta-analysis to evaluate the efficacy of ERBT in treating NMIBC by integrating DM presence rate in primary ERBT specimens and tumor residual and upstaging rate in reresection specimens. In addition, we also compared the prognostic indicators of the reresection and non-reresection groups to assess whether patients would benefit from reresection. We believe that if the efficacy of ERBT is satisfactory and the patient cannot derive sufficient benefit from reresection, an attempt can be made to avoid reresection appropriately.

## Methods

### Search strategy

We conducted research in PubMed, EMBASE, Cochrane Library, and Web of Science up to October 12, 2021, to identify studies on reresection after initial ERBT. The search terms used include: (“bladder neoplasm” OR “bladder cancer” OR “bladder tumor” OR “carcinoma of bladder”) and (“en bloc” OR “en-bloc” OR “en-bloc”) and (“second” OR “repeat” OR “reresection” OR “restaging” OR “reTUR”). We also scanned references of key articles and searched the grey literature to ensure we did not miss any relevant articles. We reported the study according to the preferred reporting items of the systematic review and meta-analysis (PRISMA) (15).

### Inclusion and exclusion criteria

Inclusion criteria are as follows: (P) patients diagnosed with primary high-grade Ta (TaHG) or T1 NMIBC who have received initial ERBT; (I) reresection performed within 12 weeks after initial ERBT; (C) no reresection after initial ERBT; (O) outcome indicators should include at least one of the following: detrusor muscle presence rate in primary ERBT specimens, tumor residual rate in reresection specimens, tumor upstaging rate in reresection specimens, comparison of prognostic data between reresection and non-reresection groups; and (S) observational study (prospective or retrospective).

Exclusion criteria are as follows: (a) case reports, comments, conference abstracts, and republished literature; (b) no interest outcome; and (c) data incomplete or invalid.

### Selection process and data abstraction

The authors first read the titles and abstracts to conduct a preliminary literature screening. Documents that meet the inclusion and exclusion criteria will be directly included in the full-text evaluation. During the full-text evaluation phase, disputes were settled by two authors through consultation. If no agreement can be reached, a third author was consulted.

Two authors independently extracted data using a predesigned data extraction table. Baseline data included the following: first author and publication year, country, study type, ERBT method, reresection cases, and reresection time. Clinicopathological data included the following: the stage and grade of the primary tumor, primary tumor size, number of primary tumors, location of the residual tumor, follow-up, and prognosis. Data required for meta-analysis included the following: detrusor muscle presence rate in primary ERBT specimens, tumor residual rate in reresection specimens, tumor upstaging rate in reresection specimens, and comparison of prognostic data between reresection and non-reresection groups.

### Literature quality and risk of bias assessment

We assessed the quality of literature using a Methodological index for nonrandomized studies (MINORS). The first eight items of MINORS were specially used for quality assessment of noncomparative studies, with 16 points. A score greater than or equal to 12 points was considered moderate to high literature quality (16).

## Statistical analysis

All statistical analyses in this study were performed using R software and Cochrane Review Manager 5.3 (China). The significance level was *P* < 0.05. In a meta-analysis of prevalence, if the event incidence was greater than 0.8 or less than 0.2, the double arcsine method will be used (17). Inconsistencies (*I*^2^) statistics were used to assess heterogeneity. *I*^2^ > 50% indicates that the heterogeneity is very significant, and the random-effect model should be adopted. *I*^2^ < 50% indicates that the heterogeneity is acceptable, and the fixed-effect model should be adopted. If heterogeneity was significant, sensitivity analysis and subgroup analysis will be used to explore the source of heterogeneity. Egger's test was used to evaluate publication bias quantitatively. *P* > 0.05 indicated no significant publication bias.

## Results

### Basic characteristics and quality assessment

**Figure 1:**
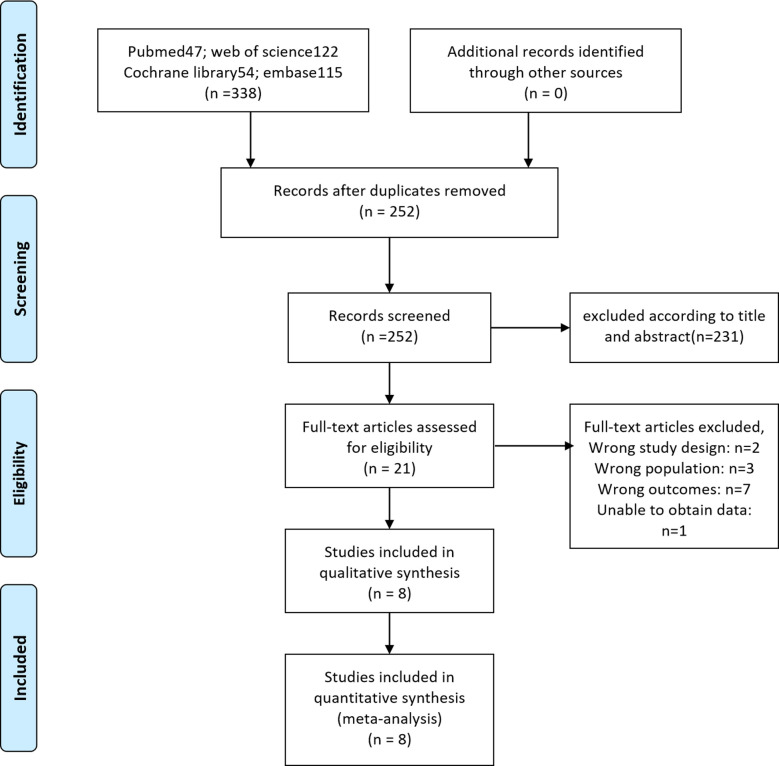
Literature search and selection.

A PRISMA flow diagram visually illustrated the screening process (Figure 1). At last, eight studies (5–9, 18–20), including 414 participants, were included by carefully screening 252 articles. Among them, five (7, 8, 18–20) were prospective and three (5, 6, 9) were retrospective. In addition, five studies (5, 6, 18–20) were laser-based ERBT, two (7, 8) were based on electrotomy, and one (9) was based on laser or electrotomy (Table 1). The clinicopathological features of patients with reresection are presented in Table 2. The MINORS scale showed that all included studies had scores greater than or equal to 12 points, and the quality of the literature was satisfactory (Table 3).

**Table 1:** Literature basic information and literature quality evaluation results.

Study	Country	Study type	ERBT method	Reresection cases	Reresection time	Outcomes	Quality scores
Wolters 2011	Germany	PS	Thulium laser	5	6 weeks	ABC	12/16
Muto 2014	Italy	PS	Thulium laser	49	30–90 days	ABC	13/16
Migliari 2015	Italy	PS	Thulium laser	53	90 days	ABC	14/16
Hurle 2020	Italy	RS	Thulium laser/Electrotomy	78	40 days	ABC	13/16
Soria 2020	Italy	PS	Electrotomy	42	2–6 weeks	ABC	14/16
Yang 2020	China	PS	Electrotomy	28	2–6 weeks	ABC	14/16
Zhou 2020	China	RS	Thulium laser	108	2–6 weeks	ABCD	14/16
Xu 2021	China	RS	RevoLix 2-µm laser	51	2–6 weeks	ABCD	13/16

PS, prospective study; RS, retrospective study; A, detrusor muscle presence rate in ERBT specimens; B, tumor residual rate in reresection specimens; C, tumor upstaging rate in reresection specimens; D, comparison of prognostic data between reresection and non-reresection groups.

**Table 2:** Clinicopathological features of patients with reresection.

Study	Initial resection results			Reresection results	Follow-up and prognosis
	T state and grade	Tumor diameter (cm)	Single lesion	Location of the residual tumor	
Wolters 2011	TaG1:1 (20%); TaG2:1 (20%); T1G3:3 (60%)	<3 (100%)	4 (100%)	0	NA
Muto 2014	TaLG:31 (63.3%); T1HG:18 (36.7%)	2.36 ± 1.47	Mixed	In situ:1	16 mon (RFS = 41/48, 85.4%; PFS = 100%);
					18mon (RFS = Ta:90%, T1:76%)
Migliari 2015	TaLG:30 (56.6%); T1HG:23 (43.4%)	2.5 (0.5–4.5)	53 (100%)	0	20mon (RFS = 46/58, 79.3%; PFS = 100%)
					18mon (RFS = Ta:90%; T1:76%)
Hurle 2020	Ta:17 (21.8%); T1:57 (73.1%); Tis:4 (5.1%); G3:72 (92.3%)	1.9 (1–3.5)	Mixed	In situ:1; Ectopic:4	30.8mon (RFS = 67/78, 85.9%; PFS = 77/78, 98.7%)
					3mon (RFS = 75/78, 96.2%)
Soria 2020	Ta:27 (64.3%); T1:8 (19.0%); Tis:7 (16.7%)	2 (1–3)	21 (50%)	In situ:1; Ectopic:1	NA
Yang 2020	HG or T1	2 (1–3)	Mixed	In situ:2	NA
Zhou 2020	Ta:60 (55.6%); T1:48 (44.4%);	2.74 ± 0.13	56 (51.9%)	NA	41.5mon (RFS = 85/108, 78.7%; PFS = 104/108, 96.3%)
	LG:25 (23.2%); HG:83 (76.8%)				12mon (RFS = 92.6%; PFS = 98.1%);
					36mon (RFS = 84.3%; PFS = 96.3%)
Xu 2021	Ta:16 (31.4%); T1:35 (68.6%)	<3 cm (42.9%)	22 (46.8%)	NA	27mon (RFS = 41/51, 80.4%; PFS = 49/51, 96.1)
	LG:13 (25.5%); HG:38 (74.5%)	≥3 cm (46.7%)			12mon (RFS = 94.1%)

LG, low grade; HG, high grade; RFS, recurrence-free survival; PFS, progression-free survival; NA, not available.

**Table 3:** MINORS assessment of included studies.

Study	MINORS criteria								
	Clearly stated aim	Inclusion of consecutive patients	Prospective collection of data	Endpoints appropriate to the aims of the study	Unbiased assessment of the study endpoint	Follow-up period appropriate to the aim of the study	Loss to follow-up less than 5%	Prospective calculation of the study size	Total
Wolters 2011	2	1	2	2	1	2	2	0	12
Muto 2014	2	2	2	2	1	2	2	0	13
Migliari 2015	2	2	2	2	2	2	2	0	14
Hurle 2020	2	2	2	2	1	2	2	0	13
Soria 2020	2	2	0	2	2	2	2	2	14
Yang 2020	2	2	2	2	2	2	2	0	14
Zhou 2020	2	2	2	2	2	2	2	0	14
Xu 2021	2	2	1	2	2	2	2	0	13

### Meta-analysis results

#### Detrusor muscle presence rate in primary ERBT specimens

**Figure 2:**
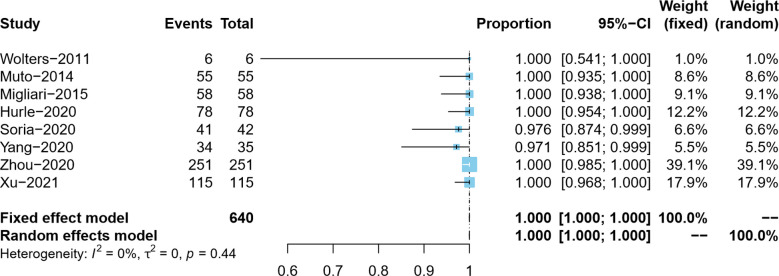
Forest plot – detrusor muscle presence rate.

Overall, the DM presence rate was reported by eight studies (5–9, 18–20). In the process of tumor resection, Yang et al. distinguished the clinical stage of bladder tumor in real-time and did not resect the detrusor muscle of the Ta tumor, so the actual DM presence rate was 97.1% (34/35) (7). Since the present rate of DM in ERBT specimens in the included studies was as high as 97.1%–100%, we adopted the double arcsine method for data conversion and, at the same time, corrected the data with the present rate of DM of 100%. Due to no pronounced heterogeneity observed (*I*^2 ^= 0%), the meta-analysis results using the fixed effects model showed that the pooled DM presence rate in the ERBT specimens and its 95% confidence interval was 100% (95%CI: 100%–100%) (Figure 2).

#### Tumor residual rate in reresection specimens

**Figure 3:**
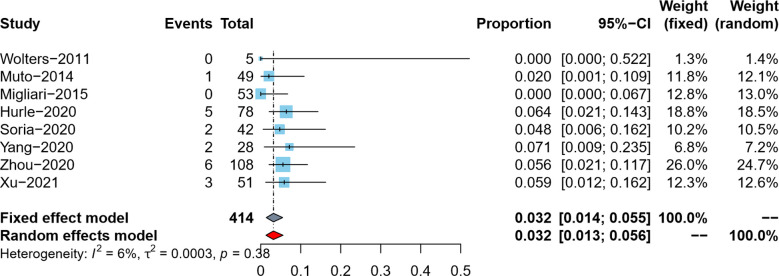
Forest plot – tumor residual rate.

Tumor residual rate was reported by eight studies (5–9, 18–20). Since the tumor residual rates in the included studies were all lower than 10%, we used the double arcsine method for data conversion, and at the same time, we corrected the data with a tumor residual rate of 0. Due to no pronounced heterogeneity observed (*I*^2 ^= 6%), the meta-analysis results using the fixed effects model showed that the pooled tumor residual rate in reresection specimens and its 95% confidence interval was 3.2% (95%CI: 1.4%–5.5%) (Figure 3).

#### Tumor upstaging rate in reresection specimens

**Figure 4:**
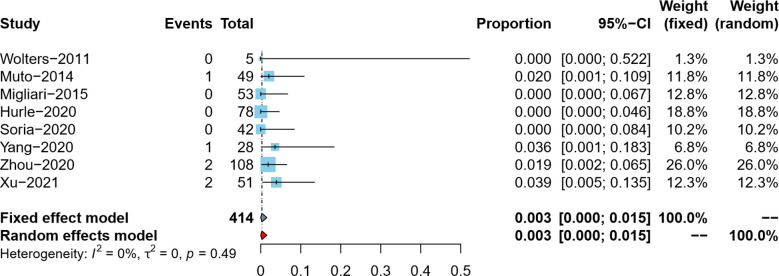
Forest plot – tumor upstaging rate.

The tumor upstaging rate was reported by eight studies (5–9, 18–20). After data conversion and correction using the double arcsine method, the meta-analysis results using the fixed effects model showed that the pooled tumor upstaging rate in reresection specimens and its 95% confidence interval was 0.3% (95%CI: 0%–1.5%) (Figure 4).

#### Comparison of prognostic data between reresection and non-reresection groups

**Figure 5:**
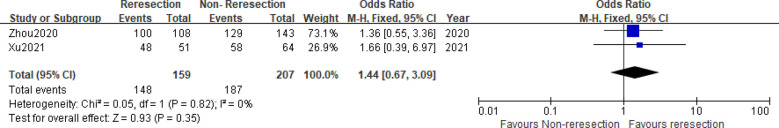
Forest plot – comparison of the 1-year recurrence-free survival rate between reresection and non-reresection groups.

**Figure 6:**
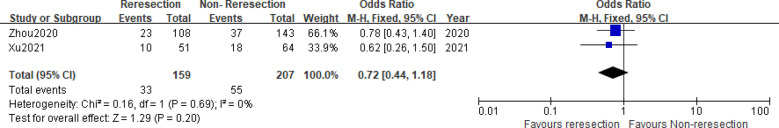
Forest plot – comparison of the tumor recurrence rate between reresection and non-reresection groups.

**Figure 7:**
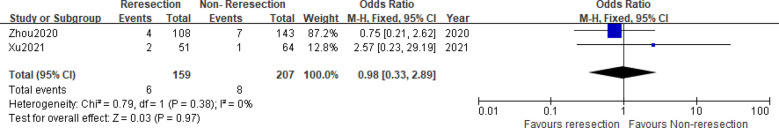
Forest plot – comparison of the tumor progression rate between reresection and non-reresection groups.

Two studies (5, 6) compared the prognostic data of the reresection and non-reresection groups after the initial ERBT (Table 4). We found no significant difference in the 1-year recurrence-free survival (RFS) rate (OR = 1.44, 95%CI: 0.67–3.09, *P* = 0.35, *I*^2 ^= 0%) (Figure 5) between the two groups nor in the rate of tumor recurrence (OR = 0.72, 95%CI: 0.44–1.18, *P* = 0.2, *I*^2^ = 0%) (Figure 6) or progression (OR = 0.98, 95%CI: 0.33–2.89, *P* = 0.97, *I*^2 ^= 0%) (Figure 7) at final follow-up.

**Table 4:** Prognosis of patients with high-risk NMIBC after initial ERBT (reresection vs. non-reresection).

Study	Groups	Initial resection result		follow-up (months)	1-year recurrence-free rate	*P*	Tumor recurrence	*P*	Tumor progression	*P*
		T stage	Grade							
Xu 2021	Reresection (*n* = 51)	Ta:16 (31.4%)	LG:13 (25.5%)	27 (5–60)	48/51 (94.1%)	0.269	10/51 (19.6%)	>0.05	2/51 (3.9%)	0.430
		T1:35 (68.6%)	HG:38 (74.5%)							
	Non-reresection (*n* = 64)	Ta:15 (23.4%)	LG:13 (25.5%)		58/64 (90.6%)		18/64 (28.1%)		1/64 (1.6%)	
		T1:49 (76.6%)	HG:38 (74.5%)							
Zhou 2020	Reresection (*n* = 108)	Ta:60 (55.6%)	LG:25 (23.2%)	40 (3–72)	100/108 (92.6%)	>0.05	23/108 (21.3%)	>0.05	4/108 (3.8%)	>0.05
		T1:48 (44.4%)	HG:83 (76.8%)							
	Non-reresection (*n* = 143)	Ta:87 (60.8%)	LG:49 (34.3%)		129/143 (90.2%)		37/143 (27.3%)		7/143 (4.0%)	
		T1:56 (39.2%)	HG:94 (65.7%)							

LG, low grade; HG, high grade.

### Publication bias

**Figure 8:**
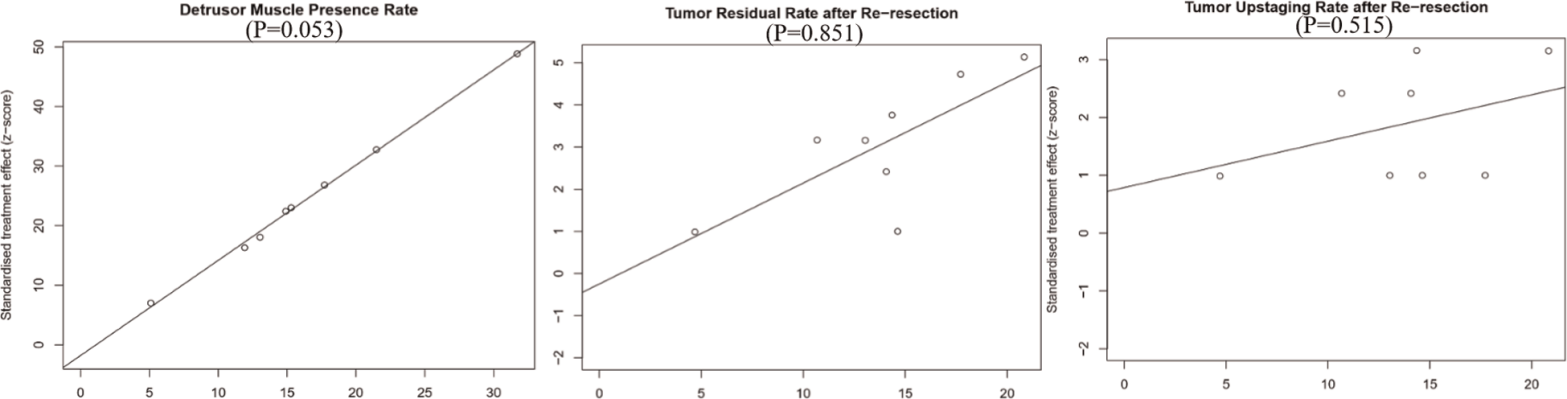
Publication bias – Egger’s graph.

We used Egger's test to evaluate publication bias quantitatively, and the results showed that no obvious publication bias was found in all outcome index groups. We showed Egger plots and *P* values for the primary outcome indicators in Figure 8.

## Discussion

To our knowledge, this study is the first meta-analysis to explore whether a reresection can be avoided for high-risk NMIBC patients after initial ERBT. For high-risk NMIBC patients who underwent traditional TURBT, the primary purposes of reresection are to improve the present rate of DM, clarify tumor stage, reduce tumor residue, and improve the prognosis of patients (21, 22). However, our study showed that the present rate of DM in primary ERBT specimens could reach 100%. On this basis, the tumor upstaging rate and tumor residual rate in reresection specimens were extremely low. A recent meta-analysis involving 29 studies also showed that ERBT had a significantly higher DM presence rate in primary ERBT specimens and a significantly lower tumor residual rate in reresection specimens than traditional TURBT. It is consistent with our study (23). In addition, our study also found that reresection did not seem to improve the prognosis of high-risk NMIBC patients with initial ERBT. It can be seen that the advantages of ERBT over traditional TURBT seem to have satisfied the original intention of carrying out reresection. Reresection after initial ERBT in high-risk NMIBC patients does not appear to be critical and essential. Considering the trauma and economic pressure brought by reresection, for patients with poor physical conditions who are difficult to tolerate reresection, it seems that an attempt can be made to avoid reresection appropriately.

When there is no DM in the initial specimen, reresection can provide detrusor muscle of the tumor bed, thus improving the accuracy of tumor staging (24). Gordon's study showed that the present rate of DM in traditional TURBT specimens was 71.2%, which increased to 87.8% after reresection (25). Han et al.'s study showed that the tumor upstaging rate was 16.1% after referring to the reresection specimens (26). A recent systematic review also showed that tumor upstaging occurred in 0%–32% (T1 to ≥T2) of cases (24). In a single-center retrospective study by Zhou et al., DM was present in all 251 ERBT participants’ specimens, and the tumor upstaging rate was only 1.9% (2/108) after reresection of 108 high-risk NMIBC patients (6). Subsequently, Xu et al. also obtained similar results in the study of 115 patients, with the DM presence rate in primary ERBT specimens and the tumor upstaging rate in reresection specimens of 100% and 3.9%, respectively (5). Our study, which integrated all available data, showed that DM was present in 100% of ERBT specimens and the tumor upstaging rate was 0.3% after referring to the reresection specimens. Regarding tumor staging, ERBT has a high presence rate of DM and excellent staging accuracy. Therefore, reresection does not seem to be indispensable in terms of tumor staging.

Cumberbatch et al. conducted a systematic review of studies on reresection after traditional TURT. For Ta tumors, the rate of residual tumors found at reresection ranged from 17% to 67%, and for T1, it ranged from 20% to 71% (24). Subsequently, the study of Akitake et al. also showed that among 143 high-risk NMIBC patients with traditional TURBT, 66 tumor residues (46.2%) were found after reresection (27). Unlike the high tumor residual rate of traditional TURBT, our study showed that patients with initial ERBT found an extremely low tumor residual rate (3.2%) at reresection. In addition, Zhou et al. and Xu et al. performed cystoscopy on patients in the non-reresection group three months after ERBT. They found that the tumor residual rate was similar to that in the reresection group (5, 6). They believe that although the cystoscopy timing differed between groups, the results may have been biased. Nevertheless, in part, it might reflect that reresection after the initial ERBT did not seem to reveal more tumor residuals than non-reresection. In summary, the tumor residual rate of ERBT is low, and reresection may not find more residual tumors. It provides a basis for avoiding reresection.

In a prospective study, patients with T1 NMIBC at initial diagnosis were randomly divided into reresection and non-reresection groups. The first- and third-year recurrence-free survival rates were 82% and 65% in the reresection group and 57% and 37% in the non-reresection group, respectively. It indicates that the reresection can significantly improve the recurrence-free survival rates of patients (28). However, the study of Calo et al. showed that if the initial resection was complete, reresection did not improve RFS and progression-free survival (PFS) in patients with high-grade T1 NMIBC (29). The study of Gontero et al. also pointed out that if the detrusor muscle was not present in the initial TURBT specimen, the RFS and PFS of T1HG patients could be improved by reresection. If the detrusor muscle was present, the patient's prognosis could not be improved by reresection (30). We believe that the mechanism of reresection to improve prognosis lies in removing the DM in the tumor bed and removing the residual tumor as much as possible. In contrast, in ERBT patients who have almost achieved R0 resection, the effect of reresection to improve prognosis will no longer be indispensable. Our results confirm this hypothesis. We found no significant difference in the 1-year RFS rate between the reresection and non-reresection group, nor in the tumor recurrence rate or progression at final follow-up. Due to a lack of data, we included only two studies, which, despite possible bias, have demonstrated to some extent that high-risk NMIBC patients with initial ERBT do not seem to obtain significant improvement in prognosis from reresection.

### Limitations

Admittedly, there are still flaws in our research. First, this study is a meta-analysis of the rate and lacks a control group, which cannot directly reflect the difference between ERBT and traditional TURBT. Second, only two studies compared the prognosis of the reresection and non-reresection groups, which is theoretically not suitable for meta-analysis. Third, due to the lack of primary data, we could not detail how many CIS, BCG nonresponse, multifocal, and 3 cm HG bladder tumors were reported in selected studies. Again, because of insufficient data, our study was not limited to T1 cases or included in subgroup analyses. Fourth, we did not consider the possibility of acquiring diabetes in sections far from the deepest part of the tumor, which may have skewed the results. Finally, despite the meta-analysis, the total sample size is still insufficient, and more large-sample randomized controlled studies are needed in the future to verify our results further.

## Conclusion

ERBT can almost completely remove the detrusor muscle of the tumor bed with very low postoperative tumor residue and upstaging rate. Reresection after initial ERBT in high-risk NMIBC patients does not appear to be critical and essential. For patients with poor physical conditions who are difficult to tolerate reresection, it seems that an attempt can be made to appropriately avoid reresection.

## Data Availability

The original contributions presented in the study are included in the article/Supplementary Material; further inquiries can be directed to the corresponding author/s.
